# Supplementary figures and images for: Intestinal Microbiota Differences in Litopenaeus vannamei Shrimp between Greenhouse and Aquaponic Rearing
Source: Life (Basel). 2023 Feb 14;13(2):525. doi: 10.3390/life13020525 (PMC9965531; doi:10.3390/life13020525)

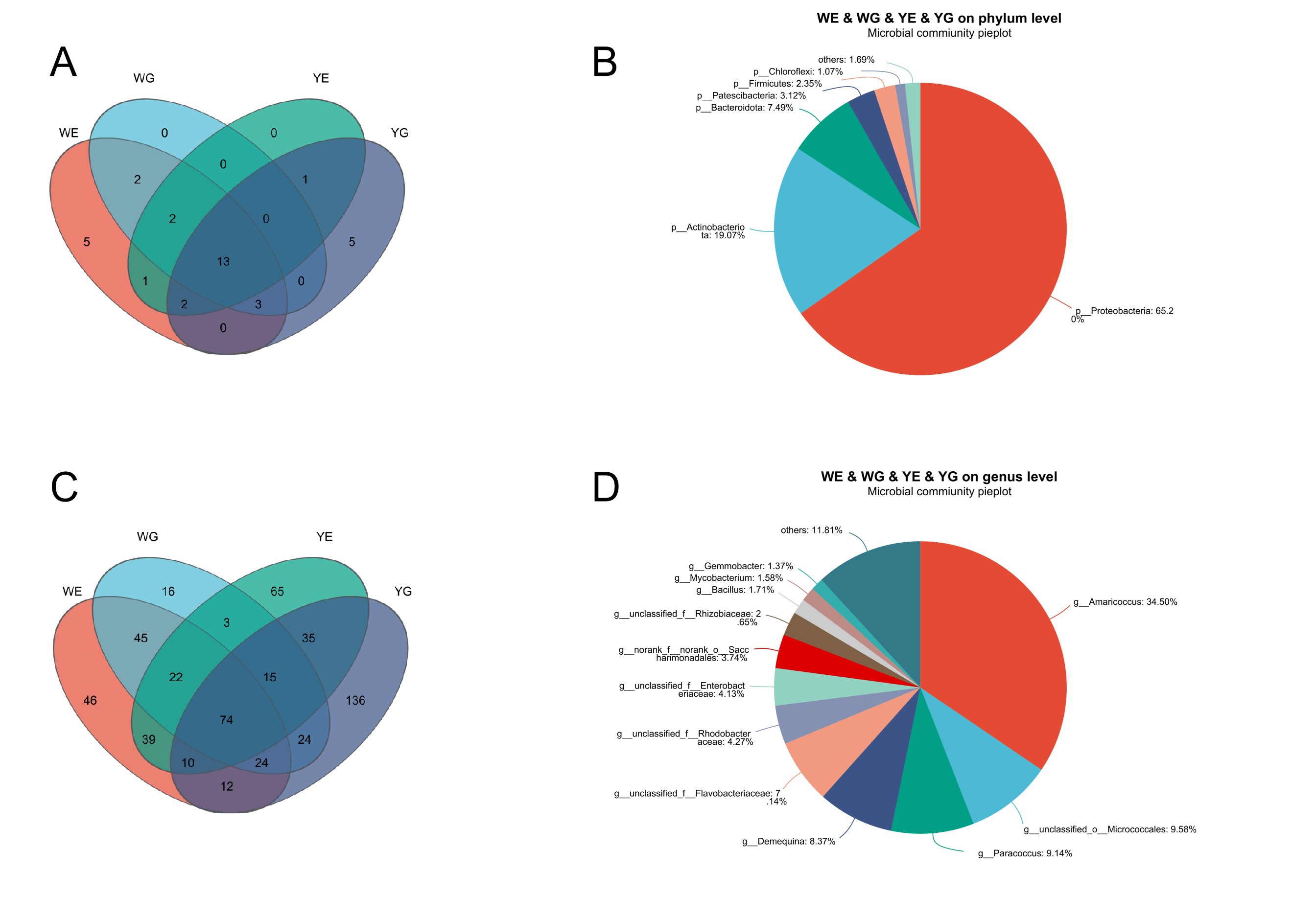

Supplement: Supplementary file 1 [file life-13-00525-s001.zip › Figure S1.jpg]
